# A Novel *RAG1* Mutation in a Compound Heterozygous Status in a Child With Omenn Syndrome

**DOI:** 10.3389/fgene.2019.00913

**Published:** 2019-10-02

**Authors:** Juan Shen, Li Jiang, Yifang Gao, Rongqiong Ou, Sifei Yu, Binyan Yang, Changyou Wu, Weiping Tan

**Affiliations:** ^1^Department of Pediatrics, Sun Yat-sen Memorial Hospital, Sun Yat-sen University, Guangzhou, China; ^2^Institute of Immunology, Zhongshan School of Medicine, Guangdong Provincial Key Laboratory of Organ Donation and Transplant Immunology, Sun Yat-sen University, Guangzhou, China; ^3^Organ Transplantation Center, The First Affiliated Hospital of Sun Yat-sen University, Guangzhou, China

**Keywords:** Omenn syndrome, RAG1 gene, SCID, mutation, Immune responses

## Abstract

Omenn syndrome is a rare autosomal recessive disorder characterized by severe, combined immunodeficiency and autoimmune features. In this case study, we found Omenn syndrome in a 3-month-old boy with recurrent infection, erythroderma, axillary lymphadenopathy, and hepatosplenomegaly. The numbers of eosinophile granulocytes and the levels of immunoglobulin E in his blood were distinctly elevated. Circulating B cells were absent, and the numbers of activated T lymphocytes were present in his peripheral blood. The production of T cell cytokines was significantly higher in the patient compared to the control samples except for interferon gamma. Whole exome sequencing revealed that the patient carried compound heterozygous mutations in the *RAG1* gene, which included a previously undescribed frameshift mutation (exon 2, 2491_2497del, p. K830fsX4) and a missense mutation (exon 2, 2923 C > T, p.R975W).

## Introduction

Severe combined immunodeficiency (SCID) is the most severe form of disease associated with primary immunodeficiency (Fischer et al., 2015; Sharapova et al., 2016). Omenn syndrome (OS) is a rare, inherited form of SCID and presents with symptoms of erythroderma, hepatosplenomegaly, lymphadenopathy, alopecia, failure to thrive, eosinophilia, hyper immunoglobulin E (IgE) levels, and the occurrence of life-threatening infections (Hönig and Schwarz, 2006; Notarangelo et al., 2016). OS is classified according to immunological phenotype, as T^+^B^−^NK^+^ or T^−^B^−^NK^+^, and is lethal unless treated with bone marrow transplantation or cord blood stem cell transplantation (Cuperus et al., 2017; Yachie, 2017).

Mutations in the recombination-activating genes 1 and 2 (*RAG1* and *RAG2*) have been reported in most OS patients and result in a deficiency of circulating B cells and nonfunctional oligoclonal T cells (Villa et al., 2001). *RAG1* and *RAG2* are, respectively, located at chromosome positions 11p12 and 11p13 and encode for the RAG1 and RAG2 proteins. RAG proteins are lymphoid-specific components of the complex of enzymes initiating the V(D)J recombination process (Fugmann et al., 2000). They play a vital role in the rearrangement process of the variable (V), diversity (D), and joining (J) segments during the development of the B and T cell receptors (BCRs and TCRs, respectively). *RAG* gene mutations cause a spectrum of severe immunodeficiencies. Based on the distinct levels of RAG expression in various patients, immunological phenotypes and clinical manifestations are diverse (Miao et al., 2018). Moreover, defects in the *Artemis* (Ege et al., 2005), *IL7R-α* (Giliani et al., 2006), *ADA* (Roifman et al., 2008), *DNA Ligase IV* (Grunebaum et al., 2009), or *CHD7* (Gennery et al., 2008) genes have been shown to be associated with OS.

Here, we present the case of a 3-month-old patient diagnosed with OS. We found a paternally inherited, previously undescribed, frameshift mutation (exon 2, 2491_2497del) on one allele of the *RAG1* gene and a maternal missense mutation (exon 2, 2923 C > T) on the other allele. Furthermore, we analyzed the clinical, immunological, and genetic characteristics of the patient in an attempt to provide information that will improve the early diagnosis and treatment of SCID or OS due to *RAG1* and *RAG2* mutations.

## Case Presentation

**Figure 1:**
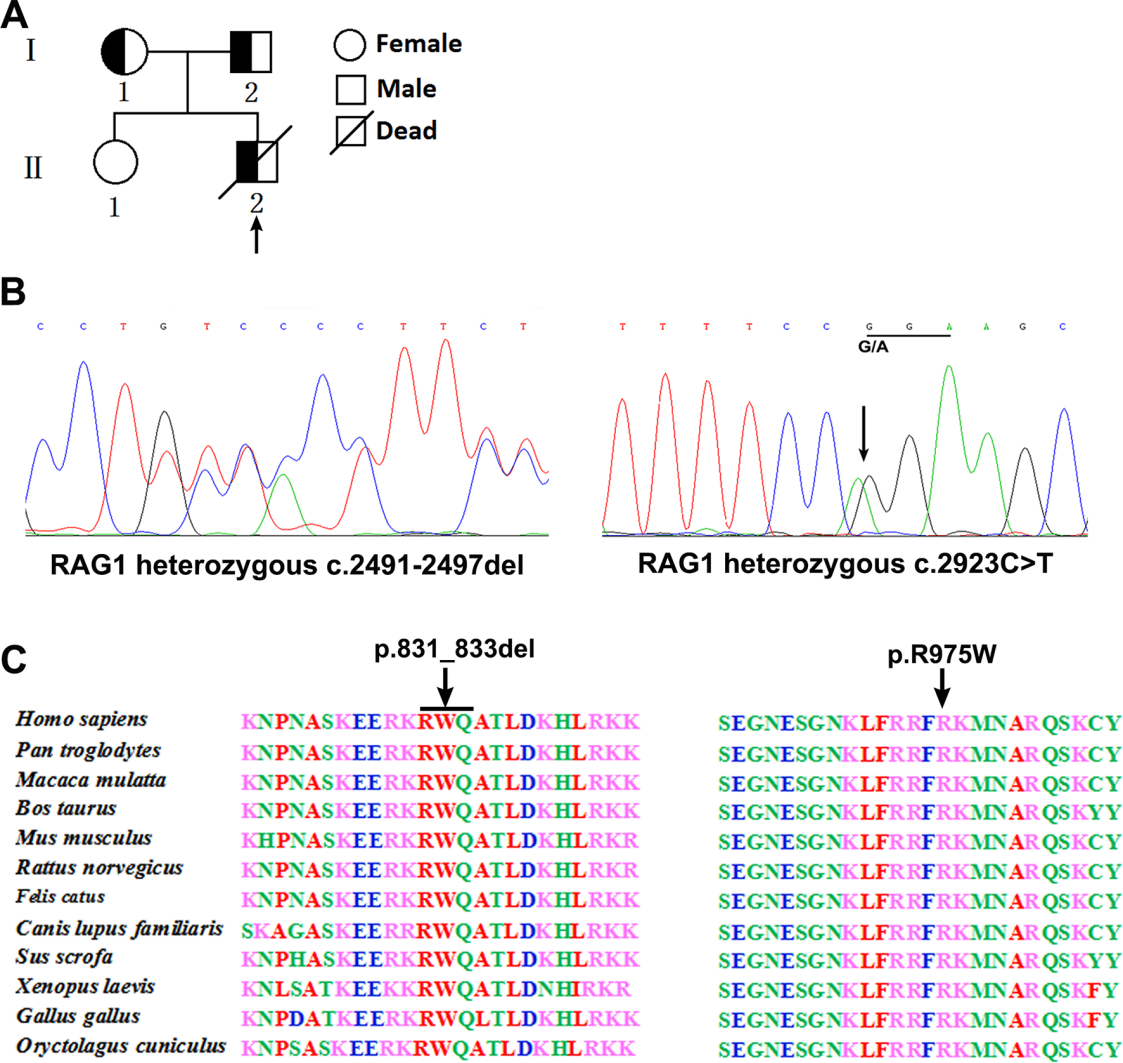
Pedigree diagrams, mutation detection, and conservation analysis. Pedigree of the family and the arrow indicates the proband **(A)**. Sequencing results showed that the frameshift mutation (c.2491_2497del) was found in the patient and his father, and the missense mutation (c.2923 C > T) was found in the patient and his mother **(B)**. Protein alignment showed conservation of the R831 and R975 residue of *RAG1* across 12 species **(C)**.

The 3-month-old boy was referred to Sun Yat-sen Memorial hospital for further therapy with the symptom of recurrent cough, prolonged fever, and axillary mass. He was the second child of healthy nonconsanguineous parents (**Figure 1A**), and born weighing 3.7 kg and had a 5-min Apgar score of 10 at full term. On admission, he was suffering from a diffused erythematous rash all over his torso. Chest auscultation revealed tachycardia and rough pulmonary breathing sounds. There was moderate hepatosplenomegaly and enlarged bilateral axillary lymph nodes with tenderness. The chest X-ray revealed pneumonia on the right side.

Laboratory examinations revealed hemoglobulin levels of 100 g/l and platelet levels of 185 × 10^9^/l. C-Reactive protein measured 82.5 mg/dl (N, < 5 mg/dl), procalcitonin was 0.2 ng/ml (N, < 0.1 ng/ml), while the erythrocyte sedimentation rate was 45 mm/h (N, < 15 mm/h). Detection of 1-3-β-D glucan and galactomannan for fungal infection were both negative as were assays for rubella, cytomegalovirus, toxoplasma, herpes, and HIV. The syphilis tolulized red unheated serum test and treponema pallidum particle agglutination assay were also negative. The purified protein derivative skin test was negative, while liver and renal function tests were normal.

Analysis of T cell receptor excision circles (TRECs) was done in the patient and his parents and compared with TREC copies in an age-matched healthy child. The TREC copies in the patient (5 copies) was significantly lower than the control group [178 copies (range, 102–319); *P* < 0.001], which is consistent with previous described (Jahnavi et al., 2019). Whole exome sequencing was performed and revealed a paternally inherited, previously undescribed frameshift mutation (c.2491_2497del, p. K830fsX4) and a missense mutation (c.2923 C > T, p.R975W) in exon 2 of RAG1 based on phenotype and genotype (**Figure 1B**). Comparison of RAG1 protein sequences across 12 distantly related animal species indicated that these mutations occurred at an evolutionarily conserved site (**Figure 1C**).

**Figure 2:**
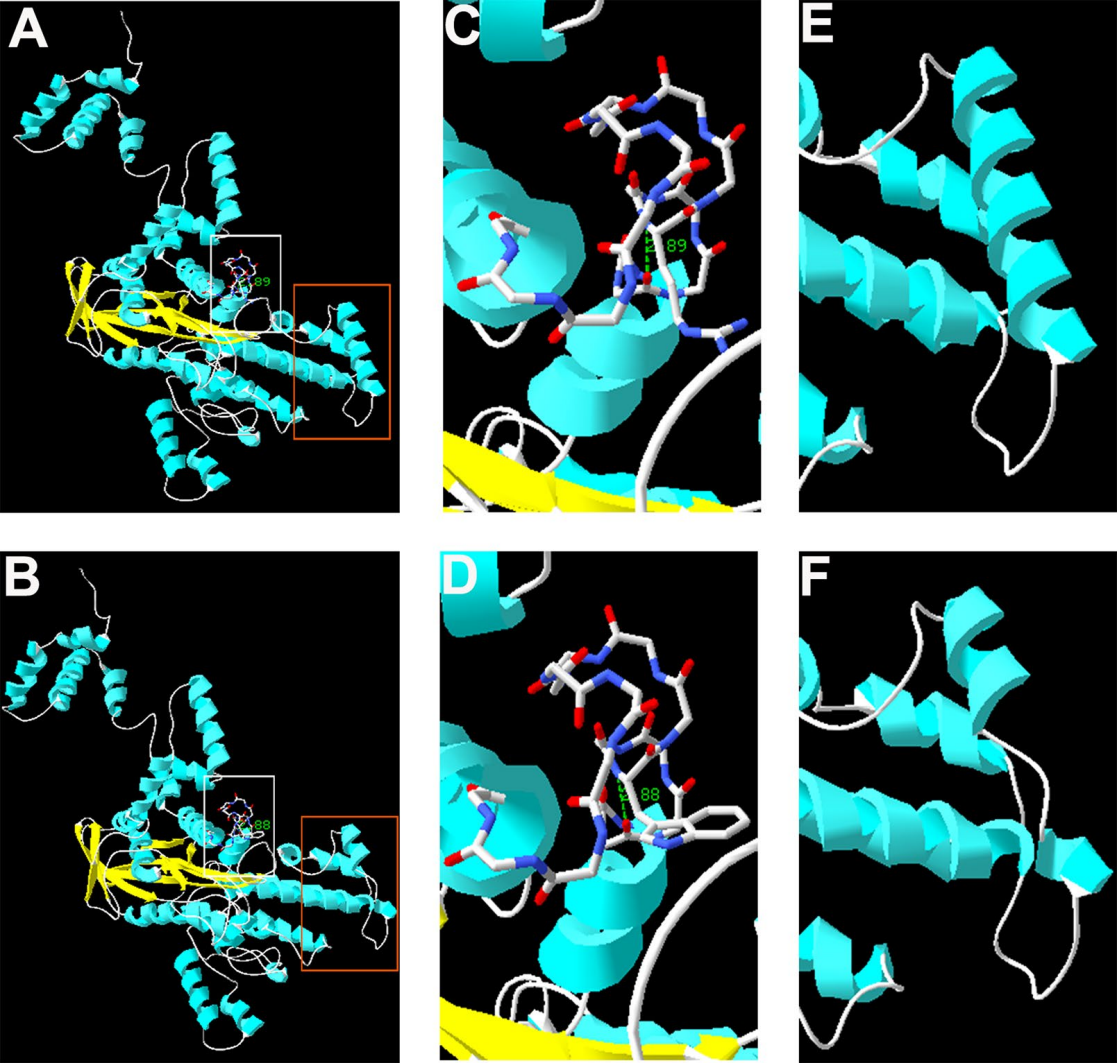
Homology modeling of wild-type and mutant *RAG1*. Modeled structures of wild-type and mutant *RAG1* protein **(A**, **B)**. Neighboring residues of R975 in the wild-type *RAG1* and 975W in the mutated *RAG1*. H bonds predicted are shown by green dashed line and Arabic numbers **(C**, **D)**. Structure of p.831_833 in wild-type *RAG1* and p.831_833del in the mutated *RAG1* **(E**, **F)**.

The complete structure of human RAG1 protein was homology modeled by Swiss-pdbViewer to predict the potential impact of each mutation on RAG1 structure. Both mutations can affect the protein structure by forming a truncated protein or by changing the hydrogen bonding distance and the spatial conformation (**Figure 2**).

## Immunological Findings

**Figure 3:**
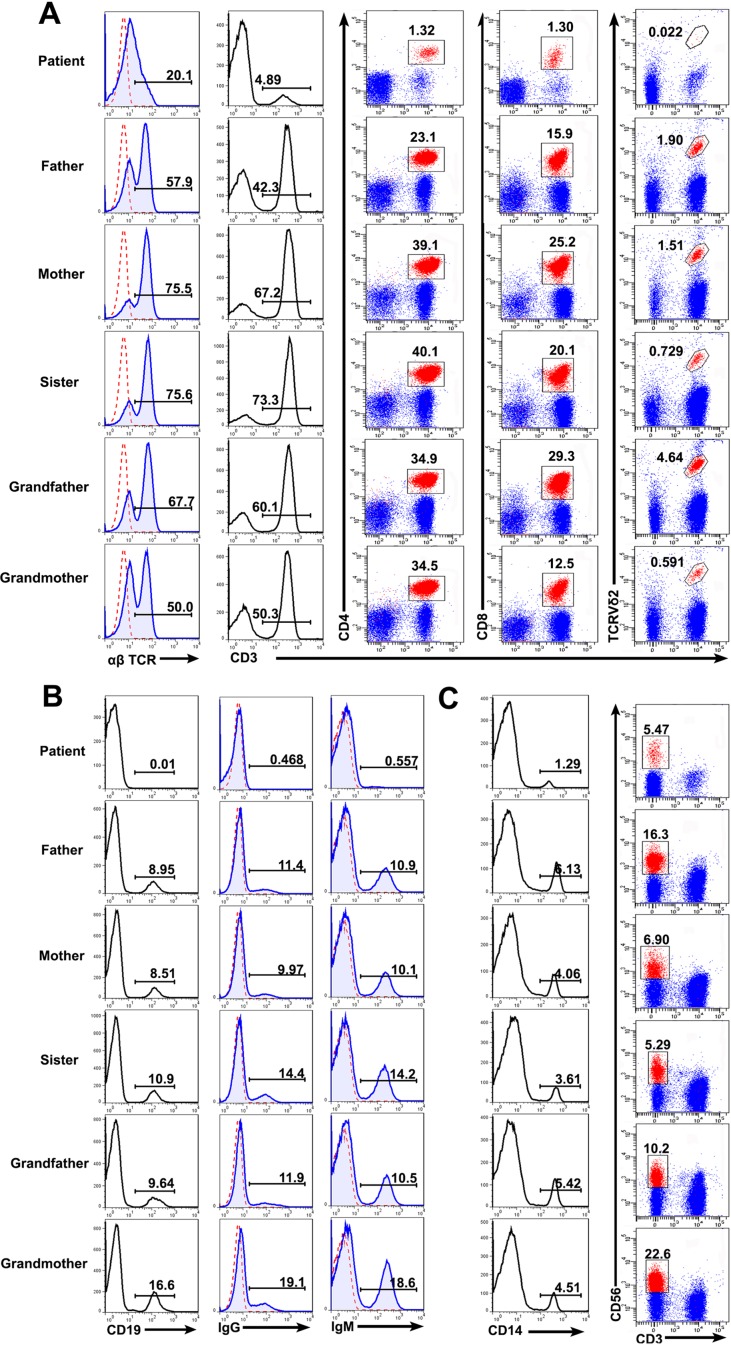
Lymphocyte counts and the expression of BCR and TCR. Peripheral blood mononuclear cells (PBMCs) were prepared from whole blood obtained from the patient and his family. The cells were stained with antibodies against CD3^+^, CD4^+^, CD8^+^, TCRVδ2^+^ T cells **(A)**, CD19^+^ B cells **(B)**, and CD3^−^CD56^+^ NK cells **(C)** and analyzed based on the gate of the lymphocytes. The cells were stained with anti-CD14 and examined by FACS **(C)**. The expression of αß T cell receptor (TCR) **(A)**, immunoglobulin G (IgG) and IgM **(B)** on the cell surface was analyzed.

Consistent with the previous report (Bai et al., 2016), the patient with *RAG* mutation had a turbulent status of lymphocytes and immunoglobulins. FACS results showed that the percentages of T cells (patient: 4.89%; controls: 42.3–73.3%), B cells (patient: 0.01%; controls: 8.51–16.6%), and monocytes (patient: 1.29%; controls: 3.61–6.13%) present in the patient were significantly lower than those from the family members. The percentage of CD3^−^CD56^+^ NK cells were comparable to one another (patient: 5.47%; controls: 5.29–22.6%). We further studied the subpopulation of T cells including CD4, CD8, and Vδ2. The percentage of CD4^+^ T cells (patient: 1.32%; controls: 23.1–50.1%), CD8^+^ T cells (patient: 1.30%; controls: 12.5–29.3%), and Vδ2^+^ T cells (patient: 0.022%; controls: 0.591–4.64%) were also affected in the patient (**Figures 3A–C**). Collectively, those data demonstrated a deficiency in the innate and adaptive immunity of the patient.

**Figure 4:**
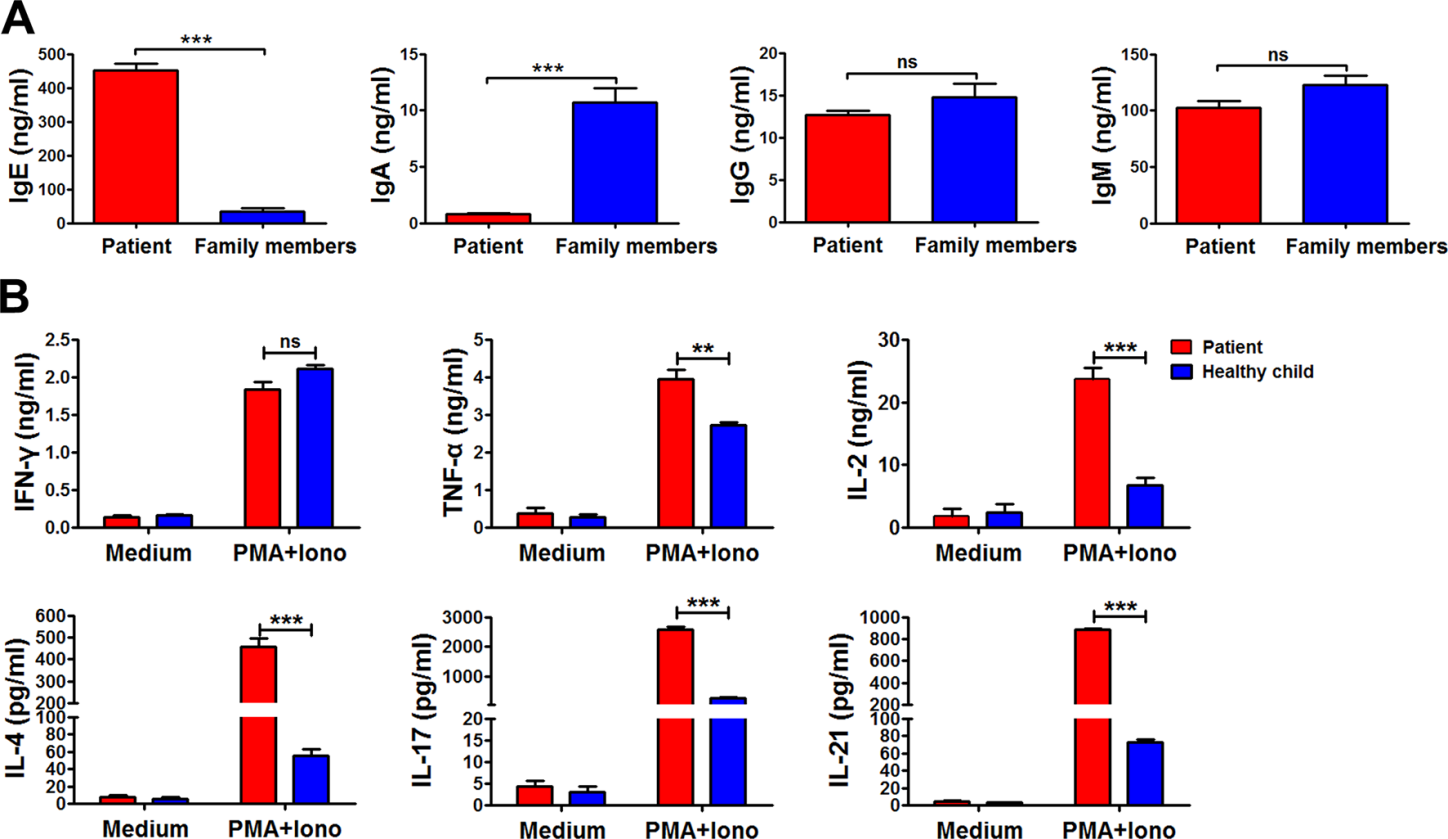
The immunological characteristics of the patient. The plasma in the patient and his family members were collected, and the levels of immunoglobulin E (IgE), IgA, IgG, and IgM in the plasma were determined by ELISA **(A)**. Peripheral blood mononuclear cells (PBMCs) from the patient and healthy child were stimulated with phorbol myristate (PMA) plus ionomycin for 24 h. The summary data showed the levels of interferon gamma (IFN-γ), tumor necrosis factor alpha (TNF-α), interleukin 2 (IL-2), IL-4, IL-17, and IL-21 in the supernatant of the cultures were detected by cytometric bead array **(B)**. Statistical significance was determined with the Mann–Whitney test (**P < 0.01; *** *P *< 0.001; ns, not significant).

Immunoglobulin expression on B cells was also investigated (Tan et al., 2015). The levels of both IgG and IgM expression were considerably lower in the patient than in other family members (**Figure 3B**). Moreover, the expression of αß TCR was substantially decreased in the patient (**Figure 3A**). Those data suggested that the mutations in *RAG1* resulted in an absence of BCR and decrease in TCR. Besides the disrupted homeostasis of different lymphocytes, humoral immunity was also variable. The plasma IgA level was strikingly decreased, but IgE was remarkably elevated in the patient, which is a typical symptom of OS (Villa et al., 2008; Notarangelo et al., 2016). Simultaneously, the plasma IgG and IgM levels were slightly decreased in the patient (**Figure 4**). Therefore, the immunological status was variably altered in OS patient.

Cytokine production by peripheral blood mononuclear cells from the patient and the healthy child was evaluated (Tan et al., 2015). Peripheral blood mononuclear cells were stimulated with or without phorbol myristate plus ionomycin for 24 h. Following stimulation, the supernatant was harvested, and the levels of interferon gamma (IFN-γ), tumor necrosis factor (TNF-α), interleukin (IL)-2, IL-4, IL-17, and IL-21 were detected by cytometric bead array (**Figure 4B**). Compared to the healthy child, production of TNF-α, IL-2, IL-4, IL-17, and IL-21, except IFN-γ, was all significantly higher in the patient (**P < 0.01; ***P < 0.001).

## Discussion

OS was described as an autosomal recessive and distinct form of SCID and was first reported by Omenn, 1965. Its pathogenesis is complicated, but mutations in *RAG1* and *RAG2* are common genetic causes of OS (Santagata et al., 2000; Miao et al., 2018). In the current study, we analyzed the clinical, immunological, and genetic characteristics of one patient with OS in our hospital. The affected boy had a frameshift mutation (c.2491_2497del, p. K830fsX4) and a missense mutation (c.2923 C > T, p.R975W) in exon 2 of the *RAG1* gene. Not having been found in the ExAc database and with a *C*-score of 223.56 and 29.2, Protein Variation Effect Analyzer score of −14.591 and −2.571, and Mutation Taster score of 1 and 0.999, respectively, both mutations were predicted to be deleterious and disease causing. Furthermore, the frameshift mutation induced a premature stop codon. Comparison of RAG1 protein sequences across 12 distantly related animal species indicates that these mutations occurred at an evolutionarily conserved site.

The patient, in this case, was diagnosed using DNA sequencing with the compound heterozygous c.2491_2497del and c.2923 C > T *RAG*1 mutations. The father was a carrier for c.2491_2497del, while the mother harbored the c.2923 C > T missense genetic variation. The c.2923 C > T *RAG1* mutation was previously identified and described by Schuetz et al. (2014). The novel c.2491_2497del mutation is associated with the truncation of the RAG1 protein. B and T cell maturation in patient was blocked, and cell quantity was decreased or became undetectable. It has to be noted that we did not estimate the rearrangement of TCR Vβ repertoires due to rapid deterioration of patient. On the basis of DNA sequencing analysis, c.2491_2497del is predicted to be a new pathogenic *RAG*1 mutation.

The analysis of phenotypes and functions of peripheral lymphocytes revealed low frequencies and dysfunctional T cells, the absence of B cells, but normal NK cell counts. Thus, the patient’s T^−^B^−^NK^+^ phenotype is known to be significantly associated with OS. In addition, eosinophil counts and IgE levels were significantly increased in the patient (Chilosi et al., 1996; Zhang et al., 2011).

Both T and B cell counts were low, repeatedly giving rise to severe infections at an early age. There was an imbalance in the Th1/Th2 ratio, thus increasing IL-4 and IL-5 secretions, and promoting the elevation of IgE. Therefore, eczema-like rashes were observed (Anna et al., 2016; Khan et al., 2017). Furthermore, the secretion IFN-γ was slightly reduced due to the decrease in Th1 cells and the increased production of Th2 cytokines. However, there was no statistical differences on IFN-γ secretion between the patient and the controls owing to the fact that Th1 cells in patient were almost effector memory T cells. Children with SCID display monoclonal TCR peaks, which are associated with T cell dysfunction. Together with B cell dysplasia, this induces cellular and humoral immune system abnormalities. Antibiotic therapy and gamma globulin treatment was inadequate and ineffective.

## Ethics Statement

All procedures performed in the study were in accordance with the ethical standards of the institutional research committee and with the 1964 Helsinki declaration. Informed consent was obtained from all individuals, and the protocol was approved by the Review Board of Sun Yat-sen University.

## Author Contributions

JS and LJ performed most experiments and wrote the manuscript; SY, RO, YG, and BY contributed to sample collection; WT and CW oversaw and designed the study.

## Funding

This study was supported by the National Natural Science Foundation of China (Grant No. 31470888) and Natural Science Foundation of Guangdong Province, China (Grant No. 6021301).

## Conflict of Interest

The authors declare that the research was conducted in the absence of any commercial or financial relationships that could be construed as a potential conflict of interest.
